# In Vitro and In Vivo Dentinogenic Efficacy of Human Dental Pulp-Derived Cells Induced by Demineralized Dentin Matrix and HA-TCP

**DOI:** 10.1155/2017/2416254

**Published:** 2017-06-28

**Authors:** Kyung-Jung Kang, Min Suk Lee, Chan-Woong Moon, Jae-Hoon Lee, Hee Seok Yang, Young-Joo Jang

**Affiliations:** ^1^Department of Nanobiomedical Science and BK21 PLUS Global Research Center for Regenerative Medicine, Dankook University, 29 Anseo-Dong, Cheonan 330-714, Republic of Korea; ^2^Department of Oral and Maxillofacial Surgery, College of Dentistry, Dankook University, 29 Anseo-Dong, Cheonan 330-714, Republic of Korea

## Abstract

Human dental pulp cells have been known to have the stem cell features such as self-renewal and multipotency. These cells are differentiated into hard tissue by addition of proper cytokines and biomaterials. Hydroxyapatite-tricalcium phosphates (HA-TCPs) are essential components of hard tissue and generally used as a biocompatible material in tissue engineering of bone. Demineralized dentin matrix (DDM) has been reported to increase efficiency of bone induction. We compared the efficiencies of osteogenic differentiation and in vivo bone formation of HA-TCP and DDM on human dental pulp stem cells (hDPSCs). DDM contains inorganic components as with HA-TCP, and organic components such as collagen type-1. Due to these components, osteoinduction potential of DDM on hDPSCs was remarkably higher than that of HA-TCP. However, the efficiencies of in vivo bone formation are similar in HA-TCP and DDM. Although osteogenic gene expression and bone formation in immunocompromised nude mice were similar levels in both cases, dentinogenic gene expression level was slightly higher in DDM transplantation than in HA-TCP. All these results suggested that in vivo osteogenic potentials in hDPSCs are induced with both HA-TCP and DDM by osteoconduction and osteoinduction, respectively. In addition, transplantation of hDPSCs/DDM might be more effective for differentiation into dentin.

## 1. Introduction

Periodontal disease and dental caries cause periodontal bone defects, which will lead to tooth loss. The best way to treat periodontal bone defects is the induction of osteogenesis through reconstructive surgery. Recently, bone tissue engineering technology is known to be a promising clinical application for replacing wounded dental tissues [1–5]. Bone regeneration requires three components: stem cells, biological factors that can undergo and enhance osteogenic differentiation, and scaffolding materials that should be biocompatible and biodegradable [3]. Mesenchymal stem cells (MSCs) are regarded as a promising source for scaffold-based bone tissue engineering due to their self-renewal and osteogenic differentiation potentials [6–8]. Various dental tissues contain the populations of MSCs, and dental stem cells are identified from dental pulp tissue, periodontal ligament, apical papilla, and dental follicles [9–12]. Unlike other MSCs, dental stem cells including human dental pulp stem cells (hDPSCs) have the advantages of nonsurgical tissue collection and easy culture, which are essential for adult stem cell-based amplification. Of these, hDPSCs contain abilities of proliferation and multiple differentiations, and they have been applied for regeneration of dentin, periodontal ligament, and bone tissue in the oral maxillofacial regions [13–16]. At early stage of osteogenic differentiation, DPSCs have shown higher alkaline phosphatase activity compared to bone marrow-derived MSCs in osteogenic medium [17]. DPSCs can be differentiated by osteoinductive bone factors such as bone morphogenetic proteins in both in vitro and in vivo studies [18–22]. However, using stem cell-based techniques for mature bone formation in large defect without additional cytokine or growth factor is still controversial, although they have potential for osteogenic differentiation. Possible reasons for conflict in stem cell therapy and tissue engineering might be a desertion of stem cells from the transplantation post and their heterogeneous differentiation properties. Inorganic-based scaffolds or carrier materials such as demineralized bone matrix, calcium phosphate scaffold, and hydroxyapatite-tricalcium phosphates (HA-TCP) have been used as platforms for cell delivery due to their osteoconductivity. In addition, they can serve as reservoirs for bioactive molecules. HA-TCP has excellent biocompatibility because their chemical and structural compositions are similar to those of inorganic-based components of natural bone tissue. These inorganic-based scaffolds have osteoconductivity and bone-bonding affinity site to enhance osteoblast differentiation and host bone cell recruitment. Nevertheless, inorganic-based scaffolds have limitations in that they have low mechanical process and that they lack bioactive molecules for osteogenic differentiation from intact host tissue [23–29]. There has been increasing interest in the development of demineralized or decellularized natural materials simultaneously having bioactive function as a solution to these problems. Many studies have been undertaken to investigate whether biological bone or dentin matrix with excellent biocompatibility and osteoconductivity could be used as biomaterial for stem cell attachment, proliferation, and osteogenic differentiation. Previous studies have shown that demineralized dentin matrix (DDM) is composed of approximately 55% inorganic minerals and 45% organic materials such as growth factors and cytokines. Based on the biochemical composition, DDM originated from animal induces bone formation potential in subcutaneous and intramuscular chambers in rodents [30] and has been used to treat bone defects by enhanced bone formation [31, 32]. DDM of enamel matrix derivative has been successfully used in clinics as bone graft and repair materials for several decades [33–36]. Biocompatible and bioactive materials for the purpose of bone tissue regeneration have been studied. Here, we show a method to isolate and culture human DPSCs and use them for in situ differentiation using inorganic scaffolds for ectopic bone regeneration without requiring additional stimuli. The first purpose of this investigation is to evaluate the osteoconductive activity of HA-TCP or osteoinductive human DDM in vitro with hDPSCs. The second purpose is to determine the abilities of hDPSCs with HA-TCP or human DDM to induce ectopic bone formation after transplanting them subcutaneously into athymic mice in vivo.

## 2. Materials and Methods

### 2.1. Culture of Human Dental Pulp Stem Cells and Transplantation Preparation

For primary culture of human dental pulp stem cells (hDPSCs), human third molars were collected from patients who were 15 to 27 years old under guidelines approved by the Institutional Review Board (IRB) of Dankook Dental Hospital (DKUDH IRB 2016-12-005). Human dental pulp tissue was obtained from the internal part of the tooth, chopped into small fragments, and enzymatically digested with 3 mg/mL collagenase type I (Millipore) and 4 mg/mL dispase (Sigma) at 37°C for 1 h. Cell suspension was incubated in *α*-MEM (HyClone) containing 20% fetal bovine serum (FBS, HyClone) and 1% antibiotics (Lonza) at 37°C in humidified atmosphere supplemented with 5% CO_2_. To observe osteoinduction efficiency in vitro, 40,000 cells per well were cultured with 0.8 g of HA-TCP (0.5–1 mm, Q-Oss+, OSSTEM Implant) or DDM (0.5–1 mm, Korea Tooth Bank, Korea) in a hanging insert cell culture dish (SPL Life Sciences). For transplantation, 1 × 10^6^ cells were resuspended in 100 *μ*L of thrombin, and 40 mg of HA-TCP or DDM granules was added. Then, 100 *μ*L of fibrinogen (TISSEEL, Baxter AG) was added and mixed immediately to allow polymerization at room temperature. Fibrin blocks of hDPSCs/HA-TCP or DDM were incubated in culture at 37°C in humidified atmosphere supplemented with 5% CO_2_ till transplantation.

### 2.2. Characterization of HA-TCP and DDM

The crystallographic properties of HA-TCP and DDM were investigated to use X-ray diffraction (XRD, UltimaIV, Rigaku, Tokyo, Japan) measurement. The XRD was activated at 40 kV and 40 mA with Cu K_*α*1_ radiation (*λ* = 1.5405 Å). The patterns of XRD were measured for step-scan of 0.02° and rate of 3° per minute over a 2*θ* range from 20 to 80°. Surface morphologies of HA-TCP and DDM were examined by scanning electronic microscopy (SEM, JSM-6510, JEOL Co., Tokyo, Japan). The HA-TCP and DDM were sputter-coated (Sputter Coater 108 Auto, Cressington, Watford, UK) with platinum and sliced to a thickness of 5 nm prior to SEM measurement. The pore size of DDM in SEM images was evaluated to use ImageJ software (NIH, Bethesda, MD, USA).

### 2.3. Preparation of DDM Extract and Western Analysis

Each HA-TCP (0.7 g) and DDM (0.7 g) granule was incubated with *α*-MEM (HyClone) without FBS at 37°C for 3 days to extract their components. Extracts were then concentrated. To detect cytokines and growth factors released from each inorganic granule, proteins in extracts were separated by SDS-PAGE, transferred to PVDF membrane, and probed with anti-collagen type-1 antibody (Santa Cruz Biotechnology) followed by incubation with horseradish peroxidase- (HRP-) conjugated secondary antibody. Protein signals were visualized by using ECL™ reagent (GE Healthcare).

### 2.4. Live/Dead Cell Survival Assay

Cell viability was determined using calcein-AM staining solution (Thermo Scientific). The fibrin blocks of hDPSCs/HA-TCP and hDPSCs/DDM were incubated in culture media for 4 days, washed with PBS, and incubated with 4 *μ*M EthD-1 and 2 *μ*M calcein-AM in PBS at room temperature for 45 min in the dark. The fluorescent signals were detected by using confocal microscope (LSM700, Carl Zeiss).

### 2.5. Subcutaneous Implantation of Fibrin Constructs in Immunocompromised Nude Mice for Ectopic Mineralized Tissue Formation

Twenty immunocompromised nude mice (female, 4 weeks old, Orient Bio, Korea) were used in this study. Animal study protocol was approved by the Institutional Animal Care and Use Committee of Dankook University. Two experimental groups were used in this study. Each group had 10 animals. Group 1 was used for fibrin gel with HA-TCP granule with or without hDPSCs. Group 2 was used for fibrin gel with DDM granule with or without hDPSCs. For transplantation, mice were anesthetized by intraperitoneal injection of a mixture of tiletamine/zolazepam (30 mg/kg, Zoletil 50®, Virbac) and xylazine (10 mg/kg, Rompun®, Bayer Korea Ltd.). Two subcutaneous 1 cm incisions were made in each flank pocket from the dorsal midline of mice. Prepared fibrin gels with granule with or without hDPSCs were placed into each pocket. After 1 week and 8 weeks, animals were sacrificed and implants were retrieved for further studies.

### 2.6. Microcomputer Tomography Examination (Micro-CT)

Mineralization and bone formation were evaluated by micro-CT. Micro-CT images were obtained using a micro-CT scanner (SkyScan-1176, Skyscan) at a resolution of 15 *μ*m pixel with a 0.5 mm aluminum filter and a rotation step of 0.4. Three readings were obtained for each sample. Bone volume was determined using a CT analyzer program (CT-An, Skyscan). 3D images were obtained using a 3D-visualization program. Statistical analyses for three readings were carried out using Student's *t*-test. Statistical significance was considered when *p* value was less than 0.05. Data are expressed as means with error bars representing standard error of the mean (SEM). Student's *t*-test was performed using GraphPad Prism 6 program.

### 2.7. Quantitative Real-Time Reverse-Transcription Polymerase Chain Reaction (qRT-PCR)

Total RNAs of hDPSCs and frozen tissue were extracted by using Easy-spin™ Total RNA Extraction kit (Intron, Korea) and RNA Easy Mini Extraction kit (Qiagen), respectively. cDNA was synthesized from total RNA by using the ReverTra Ace™ qPCR RT kit (Toyobo Corporation), and the qRT-PCR was performed by using iTaq™ Universal SYBR™ Green Supermix (Bio-Rad) system. Used primers are listed in Table 1. The cycling parameters of qPCR were followed; 1 cycle for 30 sec at 95°C, 40 cycles for 15 sec at 95°C, and 1 minute at 55°C–64°C. During PCR, a dissociation curve was constructed in the range of 65°C to 95°C.

GAPDH was used as an internal control to normalize the variability in target gene expression. Statistical analyses on three readings were carried out using Student's *t*-test and *p* values of less than 0.05 were considered statistically significant. Data are expressed as means (*n* = 3) with error bars representing standard error of the mean (SEM). Student's *t*-test was performed using GraphPad Prism 6 program.

### 2.8. Histological Analysis

After 8 weeks, the retrieved samples from dorsal midline of athymic nude mice were fixed in 4% paraformaldehyde (PFA) overnight. The fixed samples were rinsed with PBS for removing residual PFA and dehydrated using 70% ethyl alcohol. And then, the dehydrated samples were decalcified using decalcifying solution-Lite (Sigma-Aldrich, St. Louis, MO, USA) for 16 hrs and embedded in paraffin wax. The embedded samples were transversely cut to obtain different cross-sectional images. The samples were sectioned with thickness of 6 *μ*m by a microtome (RM2255, Leica, Bensheim, Germany). Sectioned samples were deparaffinized in xylene, hydrated with a series of graded ethanol, and stained with hematoxylin/eosin and Golder's trichrome staining. An optical microscope (CKX41, Olympus, Tokyo, Japan) was used to obtain images of the stained samples for confirming newly the deposition of mineralization by HA-TCP and DDM.

### 2.9. Immunohistochemical Analysis

Sectioned samples were selected randomly (*n* = 6 per group) and immersed in xylene and preceded a preconditioning process for double immunofluorescence staining of osteogenic and dentinogenic markers with human nuclear antigen (HNA). The samples were incubated in blocking buffer for 1 hr at room temperature. After blocking, samples were stained for 16 hrs with primary human specific antibodies; a 1 : 100 dilution of antibone sialoprotein antibody (Abcam), 1 : 200 dilutions of antihuman nuclear antigen (Abcam) and antiosteocalcin antibody (Abcam), a 1 : 500 dilution of antiosteopontin antibody (Abcam), a 1 : 1000 dilution of antiosteonectin antibody (Millipore), and 1 : 100 dilution of antidentin sialophosphoprotein antibody (Abcam). Fluorescein-isothiocyanate conjugated secondary antibodies (Jackson Immuno Research Laboratories, PA, USA) and 4′,6′-diamidino-2-phenylindole (DAPI, Vector Laboratories, Burlingame, CA, USA) were used to visualize the signals of the primary antibodies and nuclei, respectively. Samples were measured by using confocal microscope (LSM700, Carl Zeiss, Jena, Germany).

## 3. Results

### 3.1. Surface Analysis of HA-TCP and DDM Scaffolds

**Figure 1:**
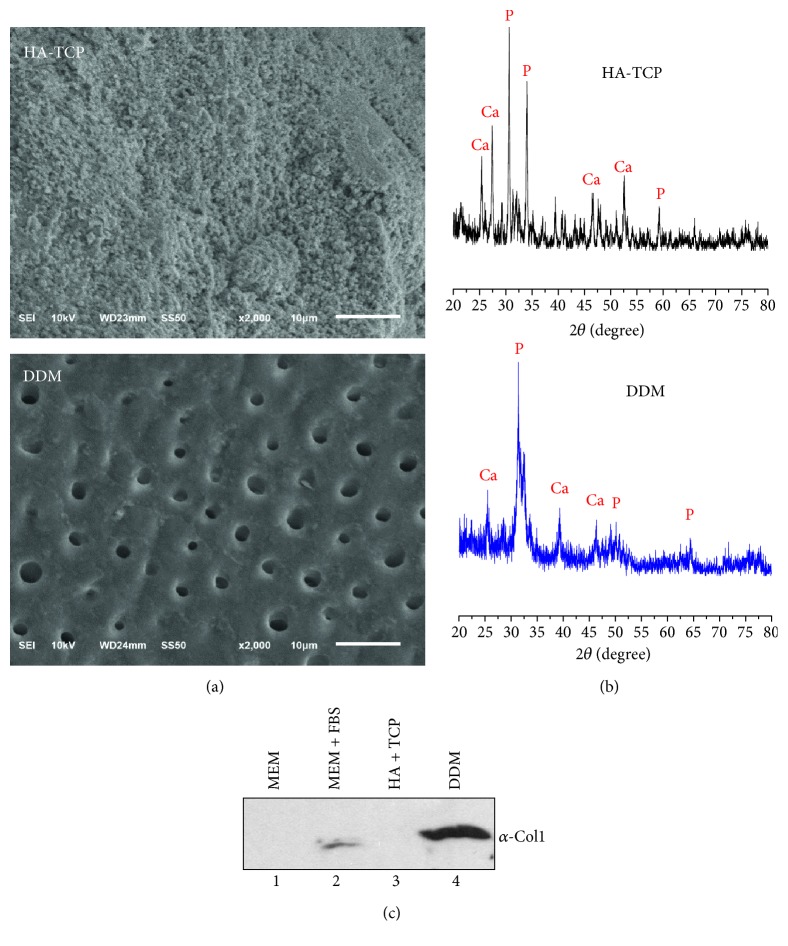
Characteristics of HA-TCP and human DDM scaffolds. (a) Surface structures of HA-TCP (upper panel) and DDM (lower panel) were examined by SEMs. Scale bars: 10 *μ*m. (b) X-ray diffraction patterns of HA-TCP (upper graph) and DDM (lower graph). (c) Analysis of protein components in materials by immunoblot. The extracts of HA-TCP and DDM granules were concentrated, separated on SDS-PAGE, and immunoblotted with anti-type-1 collagen antibody. 1, *α*-MEM only; 2, *α*-MEM with 10% FBS; 3, HA-TCP extract in *α*-MEM; 4, DDM extract in *α*-MEM.

Demineralized dentin matrix (DDM) used in this study was a congeneric biomaterial produced from human dentin for this research. It was prepared by grinding, degreasing, and decalcification (performed by Korea Tooth Bank, Seoul, Korea). Two different specimens were randomly selected to determine their pore size and surface morphologies by SEM analysis. Results of SEM analysis are shown in Figure 1. HA-TCP substrate was observed to have hydroxyapatite particles completely covering the surface of HA-TCP without pore or microtube structure (Figure 1(a), upper panel), while the surface of DDM exhibited a smear layer of enamel with continuous uniformly micro pores at diameter of 2.08 ± 0.37 *μ*m (Figure 1(a), lower panel). XRD analysis revealed several diffraction peaks produced by hydroxyapatite of HA-TCP (Figure 1(b), upper graph). Diffraction peaks at 2⊖ = 25.8 (Ca), 31.9 (P), 37.2 (Ca), 45.5 (Ca), and 47.7 (P) corresponded well to expected spectra of the hydroxyapatite. Generally, the enamel layer on the tooth consists of pure hydroxyapatite composite. Because enamel was not completely removed from the sample tooth during demineralization, hydroxyapatite components such as phosphate and calcium were detected in the DDM used in this study. Peaks of the DDM showed a typical XRD pattern of synthesized hydroxyapatite, similar to the HA-TCP sample. XRD analysis for the DDM revealed numerous peaks of Ca and P, indicating the presence of enamel on the DDM surface (Figure 1(b), lower panel). In addition to inorganic components, dentin contains organic biomolecules. To detect protein component in materials, western blot analysis was performed using DDM and HA-TCP extracts. Indeed, collagen type-1 was detected in DDM extract (Figure 1(c), lanes 4). However, they were not detected in HA-TCP extract (Figure 1(c), lanes 3). Although very weak signals of these proteins were also detected in normal culture media containing 10% FBS (Figure 1(c), lanes 2), much higher amounts of collagen were released from the DDM.

### 3.2. In Vitro Osteoinduction Potentials of HA-TCP and DDM on hDPSCs

**Figure 2:**
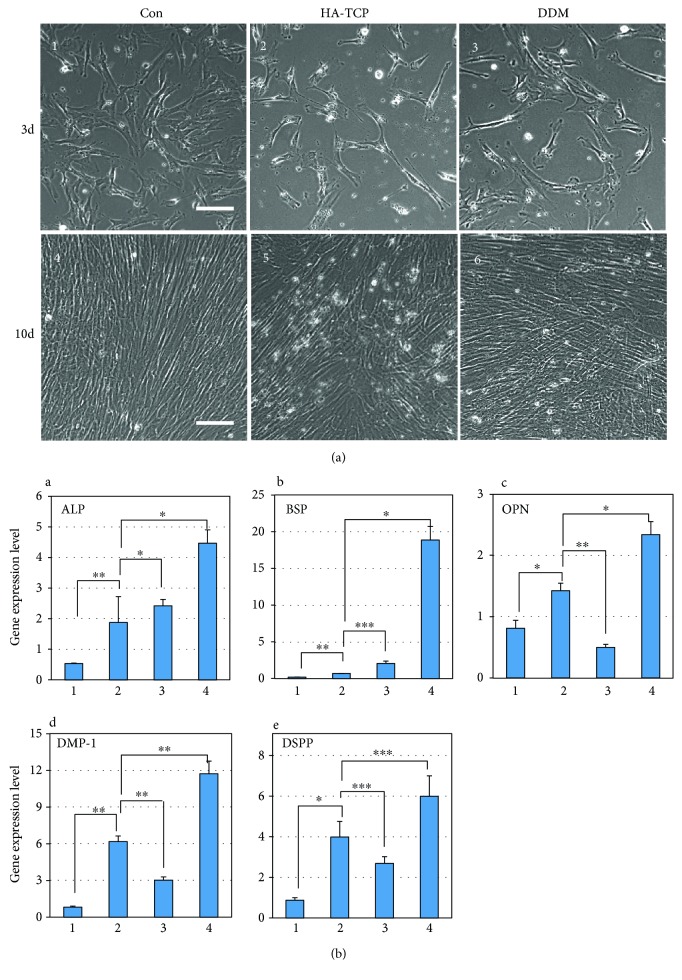
Osteoinduction potentials of HA-TCP and DDM on hDPSCs in vitro. (a) Morphology of hDPSCs cultured in the extracts of HA-TCP and DDM for 3 and 10 days. 1 and 4, hDPSCs cultured in *α*-MEM with 10% FBS, indicated as *Con*; 2 and 5, hPDSCs cultured in HA-TCP extract; 3 and 6, hDPSCs cultured in DDM extract. Scale bars: 100 *μ*m. (b) Gene expression of osteogenic and dentinogenic markers in hDPSCs cultured in the extracts of HA-TCP and DDM. Gene expressions of ALP (A), BSP (B), OPN (C), DMP-1 (D), and DSPP (E) were analyzed by the qRT-PCR. 1, actively growing hDPSCs; 2, hDPSCs in the prolonged culture for 10 days; 3, hPDSCs cultured in HA-TCP extract for 10 days; 4, hDPSCs cultured in DDM extract for 10 days. Statistical analyses were carried out using Student's *t*-test. ^∗^*p* < 0.05; ^∗∗^*p* < 0.01; ^∗∗∗^*p* < 0.001.

To determine the osteoinduction potential of HA-TCP or DDM, hDPSCs were cultured under the inserts with 800 mg of HA-TCP or DDM. Actively growing hDPSCs are generally detected as well-spreading and flat shapes (Figure 2(a), panel 1). Cell morphology was changed to a spinous and elongated phenotype, which aligned tightly and paralleled in confluent culture after 10 days (Figure 2(a), panel 4). Cells cultured with extracts of HA-TCP and DDM for 10 days also showed a similar phenotype as those under confluent culture condition (Figure 2(a), panels 5 and 6). The mRNA expression of osteogenic markers in these cells was then examined. The expression levels of alkaline phosphatase (ALP), bone sialophosphoprotein (BSP), osteopontin (OPN), dentin sialophosphoprotein (DSPP), and dentin matrix protein (DMP-1) were significantly increased in cells cultured after 10 days without addition of materials in comparison to those in actively growing cells after 3 days of culture (Figure 2(b), bars 1 and 2 in A–E), suggesting that hDPSCs could be differentiated into osteo/odontoblasts by themselves under the prolonged culture condition. Because the synthetic materials such as HA-TCP do not have osteoinduction potential, the osteo/dentinogenic markers in hDPSCs incubated with HA-TCP were not much increased in comparison to those in cells cultured for 10 days (Figure 2(b), bars 2 and 3 in A–E). Unlike HA-TCP, mRNA expression levels of osteogenic markers such as ALP, BSP, and OPN in hDPSCs with DDM extract were 2.4, 27.3, and 1.6 times higher, respectively, than those without the addition of DDM material (Figure 2(b), bars 2 and 4 in A–C). Regarding osteogenic markers, mRNA expression levels of two odontogenic markers DMP-1 and DSPP in hDPSCs cultured with DDM were 2.0 and 1.9 times higher, respectively, than those without the addition of DDM (Figure 2(b), bars 2 and 4 in D and E). These results indicate that DDM has better potential for osteoinduction in hDPSCs in vitro in comparison to synthetic hydroxyapatite materials such as HA-TCP.

### 3.3. Cell Viability of hDPSCs Cultured in Fibrin Gel Block with HA-TCP or DDM

**Figure 3:**
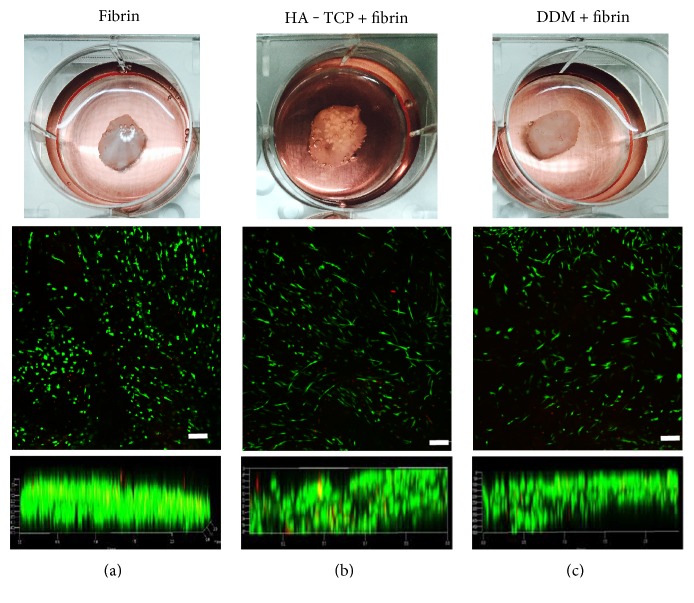
Cell viability of hDPSCs cultured in fibrin gel block. Cells were encapsulated with HA-TCP and DDM in fibrin and cultured in media for 4 days. Fibrin gen blocks were built at a certain size (upper panels). Scale bars: 1 cm. Cell viability was analyzed by using the live-dead viability assay kit. Live and dead cells were stained as green and red, respectively. Cells were detected on the surface of the block (middle panels) and in the interior of the blocks as a cross-sectional view of the block (lower panels). (a) Fibrin gel with hDPSCs. (b) HA-TCP/fibrin gel with hDPSCs. (c) DDM/fibrin gel with hDPSCs. Scale bar: 200 *μ*m.

For transplantation, hDPSCs were mixed with HA-TCP or DDM granules. To keep cells gathering in transplantation area and promote cell survival, it is necessary to encapsulate cells and biomaterial granules within fibrin gel. Culturing cells in a three-dimensional matrix is an important technique for tissue engineering as well as for studying cellular responses under culture conditions with biomaterials in vitro. hDPSCs were encapsulated with HA-TCP and DDM-fibrin gel, which had a similar size of 1 cm in width and 0.4 cm in height (Figures 3(a), 3(b), and 3(c), upper panels), and all samples were incubated in culture media. Cell viability of hDPSCs was analyzed by live and dead assay after 4 days of culture. These hDPSCs were viable inside the fibrin gel (Figures 3(a), 3(b), and 3(c)). Regardless whether HA-TCP or DDM was added, high cell viabilities were observed on the surface (Figure 3, middle panels) and in the interior of these gel constructs (Figure 3, lower panels).

### 3.4. Ectopic Bone-Forming Efficacy of hDPSCs Transplanted with HA-TCP or DDM in Subcutaneous of Athymic Mice

**Figure 4:**
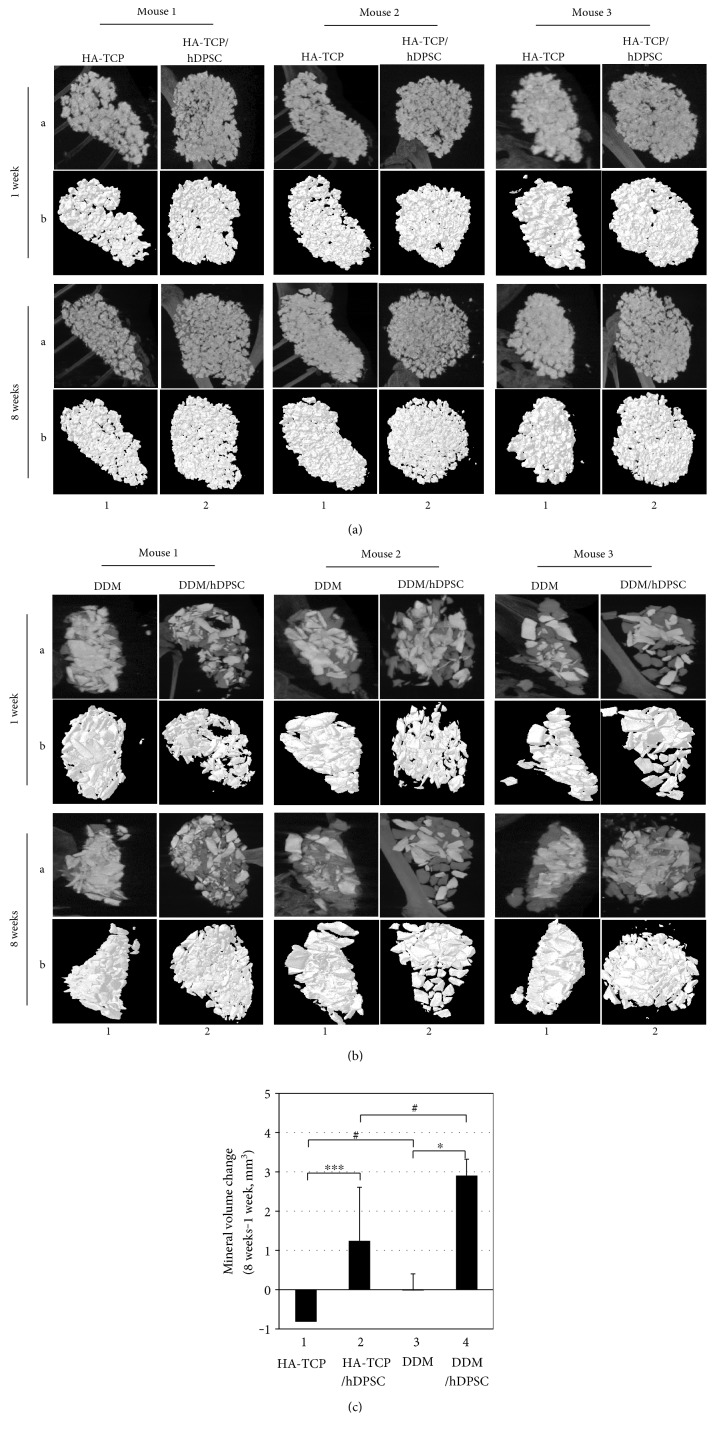
Ectopic bone-forming efficacy of hDPSCs transplanted with HA-TCP or DDM in athymic nude mice. Bone formations in the transplantation of HA-TCP (a) and DDM (b) were analyzed by micro-CT. The results were shown as X-ray images (A) and 3D-visualization image (B). (c) Quantification of mineral volume change over 1 week and 8 weeks. 1, transplantation of HA-TCP only; 2, transplantation of HA-TCP with hDPSCs; 3, transplantation of DDM only; 4, transplantation of DDM with hDPSCs. Statistical analyses were carried out using Student's *t* test. ^∗^*p* < 0.05; ^∗∗∗^*p* < 0.001; #, significant difference not be detected.

**Figure 5:**
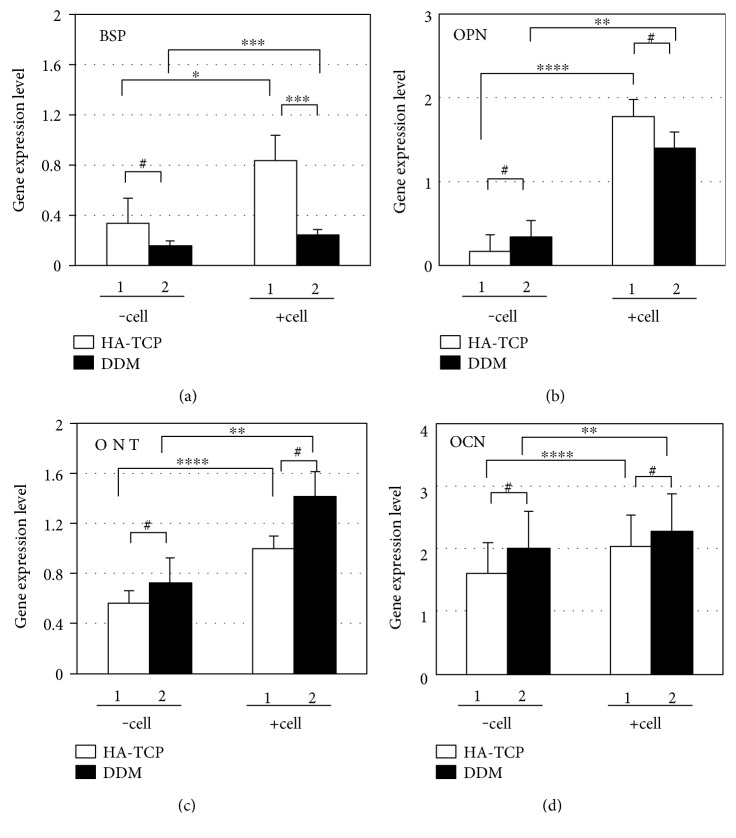
Analyses of gene expression of osteoblast markers in transplants. Fibrin blocks of HA-TCP/hDPSCs or DDM/hDPSCs were transplanted in athymic nude mice, and total RNA was isolated from transplant tissues after 8 weeks. Gene expressions of BSP (a), OPN (b), ONT (c), and OCN (d) were detected by qRT-PCR. 1, transplants with HA-TCP with or without hDPSCs; 2, transplants with DDM with or without hDPSCs. Transplantations with and without hDPSCs indicated as *–cell* and *+cell*, respectively. Statistical analyses were carried out using Student's *t*-test. ^∗^*p* < 0.05; ^∗∗^*p* < 0.01; ^∗∗∗^*p* < 0.001; ^∗∗∗∗^*p* < 0.0001; #, significant difference not be detected.

**Figure 6:**
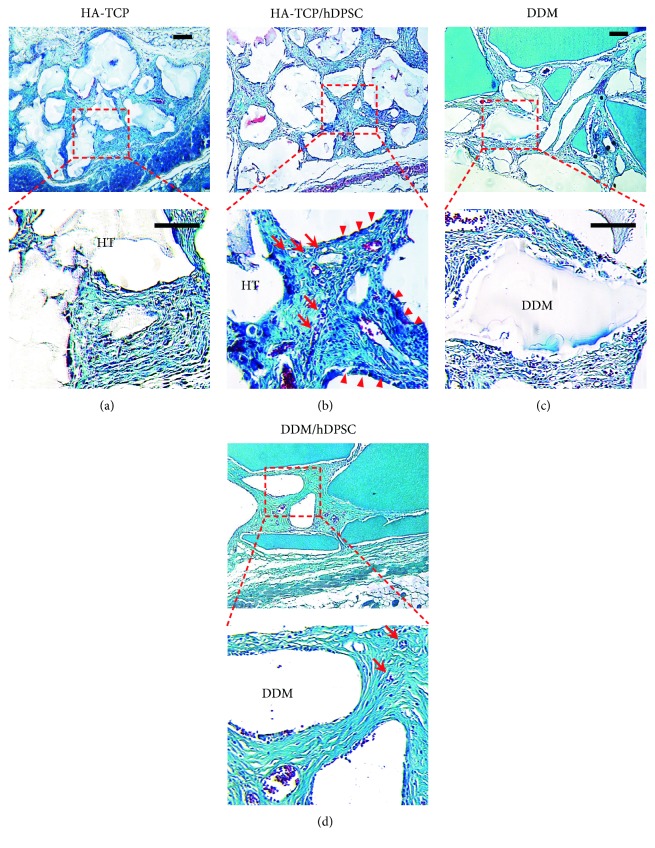
Histological analysis of bone-like tissue formed in transplants. Bone formation in vivo in athymic nude mice was examined by Goldner's trichrome staining of paraffin sections of transplants. (a) Transplants of HA-TCP only. (b) Transplants of HA-TCP with hDPSCs. (c) Transplants of DDM only. (d) Transplants of DDM with hDPSCs. Osteoid and lacuna structures were indicated as arrow heads (►) and arrows (→), respectively. Scale bar: 100 *μ*m.

**Figure 7:**
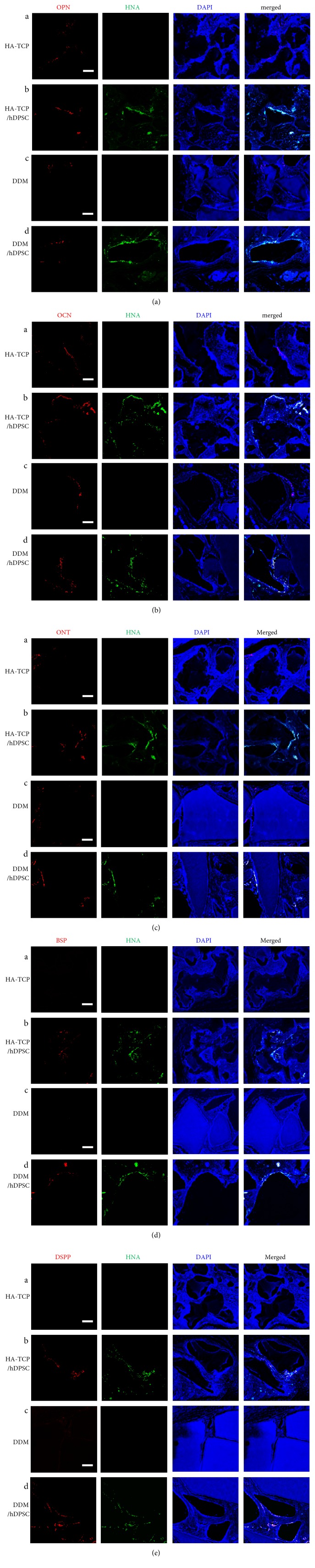
Immunohistochemical localization of osteogenic and dentinogenic markers. Paraffin sections of the transplants were incubated with anti-OPN antibody (a), anti-OCN antibody (b), anti-ONT antibody (c), anti-BSP antibody (d), and anti-DSPP antibody (e). Signal of markers was shown as red. Nuclei of human cells and DNA in tissues were stained by anti-HNA antibody (green) and DAPI (blue), respectively. (A), transplants of HA-TCP only; (B), transplants of HA-TCP with hDPSCs; (C), transplants of DDM only; (D), transplants of DDM with hDPSCs. Scale bar: 100 *μ*m.

To determine the effect of HA-TCP or DDM on ectopic bone formation of hDPSCs, cells and materials encapsulated in fibrin blocks were implanted subcutaneously into athymic nude mice. After 1 week and 8 weeks of transplantation, samples implanted on the dorsal part were analyzed directly using micro-CT. The mice were under anesthesia during the analytic process. All sample groups of HA-TCP, hDPSC/HA-TCP, DDM, and hDPSC/DDM could be apparently seen subcutaneously on the dorsal area. They all kept their initial shapes, and hard tissue formations by mineralization were detected. Micro-CT images for retrieved transplants are shown in Figures 4(a) and 4(b). The differences of bone formation between 1 week and 8 weeks were evaluated as mineral volume change. In the samples without cells, bone volumes were not increased from 1 week to 8 weeks (Figure 4(c), bars 1 and 3). When hDPSCs were encapsulated, bone volumes of the HA-TCP group and the DDM group were increased as much as 1.24 mm^3^ and 2.91 mm^3^, respectively, at 8 weeks compared to those at 1 week (Figure 4(c), bars 2 and 4). Regarding materials transplanted, gene expression of BSP and OPN were enhanced in the transplant of hDPSC/HA-TCP, which were 3.5 and 1.3 times higher, respectively, compared to those in the transplant of hDPSC/DDM (Figure 5, bars 2 in A and B). Reversely, expression of ONT and OCN were slightly enhanced in hDPSCs/DDM transplant, compared to those in hDPSC/HA-TCP transplant (Figure 5, bars 2 in C and D). Only BSP expression level was significantly increased in HA-TCP transplant (^∗^*p* < 0.001), but the expression of other genes was not significantly different in between HA-TCP and DDM. Although DDM had higher osteoinduction potential than HA-TCP, osteoconduction ability of HA-TCP could also be induced after in vivo transplantation experiment for 8 weeks. Goldner's trichrome staining results showed nearly no bone formation in implantation of HA-TCP or DDM without hDPSCs after 8 weeks of subcutaneous transplantation in mice. Implantation of HA-TCP or DDM resulted in regeneration of fibrous-like tissue (Figures 6(a) and 6(c)). However, osteoid formation around HA-TCP was observed in the transplantation group of HA-TCP with hDPSCs (Figure 6(b)). In newly formed bone (green color indicating mineralized bone), osteocytes in lacuna shape were observed in the group of HA-TCP and DDM with hDPSCs (Figures 6(b) and 6(d)). Ectopic bone formations were further supported by double immunofluorescent staining for bone-related markers and human nuclear antigen (HNA) as a marker for the detection of hDPSCs transplanted (Figure 7). A number of transplanted hDPSCs were found on the lining of the surface of coencapsulated HA-TCP and DDM granules at mineralized sites. Osteogenic marker proteins OPN, OCN, ONT, and BSP were expressed on the coincident area lined with cells (Figures 7(a), 7(b), 7(c), and 7(d)). Interestingly, DSPP, one of the dentin markers, was strongly detected in hDPSCs lined on the surface of DDM (Figure 7(e)) than those on the surface of HA-TCP transplanted. These results suggest that both HA-TCP and DDM induced in vitro osteogenic differentiation potential of hDPSCs transplanted, and they enhanced ectopic bone tissue formation. Inflammation was not observed in any histological section at the implanted site.

## 4. Discussion

Generally, human dental pulp stem cells are cultured from pulp tissues extracted from wisdom and deciduous teeth. They can be differentiated into various lineage types such as odontoblast, osteoblast, chondrocyte, adipocyte, and neural cells [22, 37–43]. To analyze osteogenesis and odontogenesis of hDPSCs, we compared inorganic based HA-TCP and human DDM under both in vitro and in vivo conditions. Primary culture of isolated hDPSCs on both biocompatible inorganic materials was performed and used in in vitro studies. For in vivo ectopic bone regeneration, both inorganic materials and hDPSCs were transplanted subcutaneously to athymic mice. Although characterization of osteoconductivity or osteoinductivity of biomaterials remains a challenge in the field of tissue engineering, osteoinductive DDM has excellent benefit for reconstructive surgery due to multiple trauma and craniofacial deformities. It is also beneficial for oncological surgery and periodontal surgery due to its osteoinductive properties. SEM analysis was performed in this study to examine surface characteristics of inorganic HA-TCP and DDM. Highly porous surface of DDM was revealed by SEM analysis, showing similarity to bony structure. The morphometrically high porous (pore size: 2.08 ± 0.37 *μ*m) DDM might also be useful as a promising scaffold in bone substitution. Furthermore, microsized pores are expected to be able to efficiently supply nutrients and oxygen in vivo (Figure 1(a), lower panel). In analysis of minerals by XRD, both HA-TCP and DDM contained large amount of calcium and phosphate (Figure 1(b)) [44]. In general thoughts, DDM should not contain minerals in its component, because they were prepared by demineralization. However, because dentin tissue used in this study for preparation of DDM was mixed with the enamel part at a certain amount, calcium and phosphate were detected in DDM even after demineralization [45]. Expression levels of osteogenic and dentinogenic markers were significantly increased in hDPSCs cultured in the extract of DDM in comparison with those in hDPSCs cultured in HA-TCP extract, suggesting that HA-TCP alone might have no effect on osteoinduction in in vitro cell culture. DDM could induce osteogenic and dentinogenic differentiation due to its osoteoinduction property (Figure 2(b)). hDPSCs transplanted with HA-TCP and DDM showed incomparable ectopic bone formation efficacy in vivo. After transplantation of HA-TCP and DDM in nude mice, both biomaterials failed to show apparent ectopic bone formation on hDPSCs after 8 weeks. Because HA-TCP itself has time-dependent resorbability without osteoinductivity [46], bone volume of HA-TCP was even slightly decreased in comparison with that of DDM. However, mineral volumes in transplants of HA-TCP/hDPSCs and DDM/hDPSCs were increased by 15.3% and by 28.7%, respectively, in comparison with those of control without cells (Figures 4(a), 4(b), and 4(c)). Expression levels of late osteogenic markers such as ONT and OCN in transplants of DDM/hDPSCs were higher than those in transplants of HA-TCP/hDPSCs (Figures 5(c) and 5(d)). Reversely, expression levels of early osteogenic markers in transplants of HA-TCP/hDPSCs were higher than those in transplants of DDM/hDPSCs (Figures 5(a) and 5(b)), demonstrating that inorganic ions released from HA-TCP could stimulate the adhesion and proliferation of marginal osteoblasts due to osteoconduction ability [47, 48]. Indeed, more osteoids were formed in transplant of HA-TCP/hDPSCs than those in DDM/hDPSCs based on histological analysis (Figure 6(b)), and lacuna structure and immature bone formed in both transplants were similar to each other (Figures 6(b) and 6(d)). Finally, the osteoconduction potential of HA-TCP in in vivo transplantation of this study seemed to be as good as that of DDM, and there was no significant difference in calcium deposition or osteogenesis after 8 weeks of transplantation between the two groups. Interestingly, the expression level of dentin-specific marker DSPP was highly detected in DDM/hDPSCs transplant, but not in HA-TCP/hDPSCs transplant (Figure 7(e)), indicating that DDM might have a better effect on dentin regeneration than HA-TCP. In conclusion, the transplantation with hDPSCs could improve the osteogenic or/and dentinogenic potential of biomaterials. This study also showed that HA-TCP and DDM might have similar effects on ectopic bone formation in in vivo animal model, although they showed the different efficacy in in vitro cellular differentiation. Additional studies are needed to determine whether hDPSC transplantation combined with osteoconduction and osteoinduction materials, which could be used in dentin regeneration. Human DDM could lead to the development of cost-effective stem cell therapy for bone tissue regeneration in clinical trials with minimal surgical operations. In addition, both biocompatible and inorganic materials might be useful for in situ osteogenic differentiation and transplantation by local delivery of hDPSCs without causing adverse effects.

## 5. Conclusion

Human dental pulp stem cells have been known to be able to form hard tissues through osteo/dentinogenesis. In current studies, HA-TCP and DDM have been used for regeneration of dentin or bone in tissue engineering. Here, we investigated the effect of osteo/dentinogenic potential of hDPSCs on DDM compared to that on HA-TCP in vitro and in vivo. Osteoinduction effect of DDM was clearly observed in vitro, but osteogenic potential was similar in both cases in in vivo transplantation. Interestingly, dentinogenic potential was detected in a higher efficacy in transplantation of DDM/hDPSCs, suggesting that DDM might be more effective than HA-TCP on dentin regeneration of hDPSCs.

## Figures and Tables

**Table 1:** Primers used for the quantitative real-time-PCR (qPCR).

Target gene	Primer sequences
Alkaline phosphatase (ALP)	For-5′-CTTGACCTCCTCGGAAGACACTC-3′ Rev-5′-CGCCCACCACCTTGTAGCC-3′
Bone sialophosphoprotein (BSP)	For-5′-TACCGAGCCTATGAAGATGA-3′ Rev-5′-CTTCCTGAGTTGAACTTCGA-3′
Osteopontin (OPN)	For-5′-GTGGGAAGGACAGTTATGAA-3′ Rev-5′-CTGACTTTGGAAAGTTCCTG-3′
Osteonectin (ONT)	For-5′-CTGTTGCCTGTCTCTAAACC-3′ Rev-5′-CACCATCATCAA ATTCTCCT-3′
Osteocalcin (OCN)	For-5′-TGAGTCCTGAGCAGCAG-3′ Rev-5′-TCTCTTCACTACCTCGCT-3′
Dentin matrix protein 1 (DMP1)	For-5′-GACTCTCAAGAAGACAGCAA-3′ Rev-5′-GACTCACTCACCACCTCT-3′
Dentin sialophosphoprotein (DSPP)	For-5′-CAGTACAGGATGAGTTAAATGCCAGTG-3′ Rev-5′-CCATTCCCTTCTCCCTTGTGACC-3'
GAPDH	For-5′-GTATGACAACAGCCTCAAGAT-3′ Rev-5′-CCTTCCACGATACCAAAGTT-3′
